# Long start strategy in high-intensity interval training in Rugby Union Academy players

**DOI:** 10.3389/fspor.2026.1858909

**Published:** 2026-06-24

**Authors:** Loïc Louit, Virgile Merlen, Quentin Bretonneau, Jonathan Ward, Olivier Gavarry

**Affiliations:** 1 Laboratory Youth-Physical Activity and Sports-Health (J-AP2S), Toulon University, Toulon, France; 2 Aviron Bayonnais, Bayonne, France; 3 Laboratory MOVE (UR20296), Faculty of Sport Sciences (UFR STAPS), University of Poitiers, France; 4 Bristol Bears, Bristol, United Kingdom

**Keywords:** endurance training, high-intensity exercise, HIIT, maximal oxygen consumption, physical fitness, team sport

## Abstract

**Introduction:**

High-intensity interval training (HIIT) protocols with varied parameters have been investigated to optimise the time spent at high levels of oxygen uptake. However, to date, the physiological and perceptual responses to a long-start approach (i.e., longer initial work phases followed by shorter subsequent phases) have not been investigated. The aim of the study was to compare external load, physiological responses, and rating of perceived exertion (RPE) between a constant work-interval HIIT protocol (HIIT-C) and a long-start strategy (HIIT-LS) in rugby union players.

**Methods:**

Nine male academy rugby union players (19.9 ± 0.8 years) from a professional team randomly completed both HIIT-C and HIIT-LS in a counterbalanced design. HIIT-C consisted of 2 × 8 repetitions of 30 s work with 15 s passive recovery and 3 min rest between sets. HIIT-LS consisted of 2 × 7 repetitions (3 × 45 s followed by 4 × 30 s), each separated by 15 s passive recovery, with 3 min rest between sets. Exercise intensity was set at 88% and 83% of maximal velocity achieved during the 30-15 Intermittent Fitness Test for HIIT-C and HIIT-LS, respectively. Differences between protocols were assessed using a paired *t*-test or, when appropriate, a Wilcoxon signed-rank test depending on data distribution.

**Results:**

HIIT-LS elicited a greater total distance and distance covered above 7 km h^−1^ compared to HIIT-C (*p* < 0.01), despite a lower mechanical work distance (*p* < 0.05). No differences were observed in time spent ≥90%V˙O_2_peak (*p* = 0.25). Perceived exertion was significantly lower following HIIT-LS (*p* < 0.05).

**Discussion:**

Time spent at high levels of oxygen uptake did not differ between HIIT-LS and HIIT-C. However, HIIT-LS was better tolerated, as reflected by lower perceived exertion, suggesting potential practical benefits for training load management. Future research should investigate the effects of incorporating HIIT-LS into long-term training interventions aimed at improving endurance performance in team-sport athletes.

## 1 Introduction

Rugby union is an intermittent collision sport, requiring repeated high-intensity efforts (e.g., accelerations, sprints, and physical collisions) interspersed with lower-intensity activities (e.g., walking and jogging) (1–5). In rugby union, the ability to repeatedly perform high-intensity actions has been strongly associated with key performance outcomes and total running distance covered during match play (6, 7), variables that become increasingly important as the level of competition rises (8). Aerobic capacity is therefore recognised as a critical determinant of an athlete's ability to sustain repeated high-intensity efforts throughout competition (7, 9). Moreover, endurance capacity has been linked to several game-specific performance indicators in rugby union, including ruck efficiency, tackling success, work rate, and overall activity rate (10, 11). Consequently, optimising endurance-oriented conditioning strategies remains a major focus of performance preparation in rugby union.

High-intensity interval training (HIIT) is a widely used method to improve endurance performance in team sport athletes (12). HIIT consists of repeated exercise bouts performed at intensities ranging from the second lactate threshold to near-V˙O_2_max, interspersed with periods of lower-intensity recovery (13–15). This training method is considered particularly time-efficient because it enables athletes to accumulate substantial physiological stress at high exercise intensities conducive to improvements in cardiorespiratory fitness (14, 16). In rugby union and other team sports, HIIT has therefore been proposed as an effective conditioning strategy due to its ability to simultaneously target aerobic development while replicating the sport's intermittent physiological and mechanical demands (12, 17, 18).

Among the physiological determinants of HIIT effectiveness, maximising time spent at or above 90% of peak oxygen uptake (T ≥ 90%V˙O_2_peak) is considered a key stimulus for promoting aerobic adaptations (19). Consequently, the manipulation of HIIT prescription variables is of considerable interest to practitioners and researchers. HIIT prescription involves the interaction of numerous parameters, including work-interval intensity and duration, recovery intensity and duration, exercise modality, number of repetitions and sets, and between-set recovery characteristics (14). Recent studies have demonstrated that varying parameters within a HIIT session, such as the fast-start HIIT strategy (HIIT-FS), in which the initial work intervals are performed at higher intensities than subsequent intervals, may optimise physiological responses with higher T ≥ 90%V˙O_2_peak compared to constant-intensity HIIT in endurance-based activities such as running (20), cycling (21–23), and cross-country skiing (24, 25), as well as in rugby union players (26). However, the practical applicability of HIIT-FS may be limited by the higher perceived exertion associated with these protocols (26), potentially reducing their tolerability and implementation in applied team-sport settings.

More recently, an alternative varied-parameter strategy involving longer initial work intervals followed by a progressive reduction in interval duration has emerged as a promising method for increasing physiological stimulus while maintaining perceptual demands. This “long-start” approach has been shown to increase T ≥ 90%V˙O_2_peak in both cyclists and runners compared with traditional long- and short-interval HIIT configurations, without significantly increasing ratings of perceived exertion (27, 28). Despite these promising findings, the effects of this strategy remain unexplored in team-sport environments, particularly in rugby union, where the intermittent locomotor profile and repeated changes in direction may substantially influence physiological and mechanical responses to HIIT.

Importantly, no previous study has investigated the effects of a long-start HIIT strategy (HIIT-LS) implemented within a rugby-specific intermittent framework based on the 30-15 Intermittent Fitness Test (30-15 IFT), a format widely used in rugby conditioning due to its strong ecological validity and relevance to intermittent team-sport demands. Furthermore, previous studies examining varied-parameter HIIT strategies have primarily focused on physiological outcomes, with limited investigation of the associated external-load and perceptual responses in applied rugby settings. Therefore, understanding whether HIIT-LS can enhance physiological stimulus without increasing mechanical or perceptual load may provide valuable practical implications for conditioning prescription in rugby union.

The purpose of this study was to compare the physiological, perceptual, and external-load responses to a long-start strategy HIIT protocol (HIIT-LS) and a constant-intensity HIIT protocol (HIIT-C) in academy rugby union players. The primary aim was to assess differences in T ≥ 90%V˙O_2_peak and mean %V˙O_2_peak between protocols. The secondary aim was exploratory and sought to compare external load variables, including total distance, distance covered at or above 7 km h^−1^, distance covered at or above 16 km h^−1^, and mechanical work distance, alongside heart rate responses and rating of perceived exertion (RPE) between conditions.

It was hypothesised that (i) HIIT-LS would elicit a greater T ≥ 90%V˙O_2_peak compared to HIIT-C, and (ii) no significant differences would be observed in perceptual or external-load responses. These hypotheses were tested at both the whole-session level and across discrete time segments within each protocol.

## 2 Methods

### 2.1 Participants

Twelve healthy male academy rugby union players volunteered to participate in this study (age: 19.8 ± 0.7 years; height: 184.1 ± 8.1 cm; body mass: 88.5 ± 11.3 kg; further characteristics reported in Table 1). Sample size was estimated *a priori* based on the primary outcome measure, T ≥ 90%V˙O_2_peak, using data from a prior study comparing HIIT-C and HIIT-FS for rugby union players performing running protocols (26). With *α* = 0.05 and a power of 0.80 (*β* = 0.20), the required sample size was estimated to range between 10 and 19 participants depending on the assumed within-subject correlation (G*Power 3.1.9.6, Universität Kiel, Germany). All players were recruited from a French first division professional rugby union club during the 2024–25 season. This study protocol was conducted within the context of routine training of players and was not in the field of health. Therefore, it did not fall within the scope of the French law on Research Involving Human Beings (Jardé Law), and formal medical ethics approval was not required. However, the study was carried out in accordance with the ethical principles of the current revision of the Declaration of Helsinki. In particular, only standard training content was implemented and no invasive procedures were performed. Following the provision of a formal information letter, all participants gave their informed consent before taking part in the study. Inclusion criteria were: age ≥18 years, participation in an academy program for a minimum of one year and not having sustained any injury in the two weeks preceding the study. Exclusion criteria were: (1) participation in another sport and (2) inability to complete all three sessions due to illness, injury, or breathing discomfort during gas analysis. Due to withdrawal resulting from illness (*n* = 2) and injury (*n* = 1), nine participants completed all three sessions and constituted the final analytic sample (age: 19.9 ± 0.8 years; height: 182.1 ± 7.4 cm; body mass: 87.4 ± 12.0 kg).

**Table 1:** Participant characteristics.

Variable	Mean ± SD
Age (years)	19.9 ± 0.8
Height (m)	1.8 ± 0.1
Weight (kg)	87 ± 12
BMI (kg m^−2^)	26.2 ± 2.5
30-15 IFT	
V˙O_2_peak (mL kg^−1^ min^−1^)	52.3 ± 5
HRpeak (bpm)	193.0 ± 7.2
V˙Epeak (L min^−1^)	173 ± 16
*f*_R_peak (cycles min^−1^)	64.2 ± 5.7
vIFT (km h^−1^)	18.0 ± 1.6

Data are presented as mea*n* ± standard deviation.

30-15 IFT, 30-15 Intermittent Fitness Test; BMI, body mass index; V˙O_2_peak, peak oxygen uptake; HRpeak, peak heart rate; V˙Epeak, peak minute ventilation; *f*_R_peak, peak respiratory frequency; vIFT, maximal running velocity achieved during the 30-15 IFT.

### 2.2 Study design

**Figure 1:**
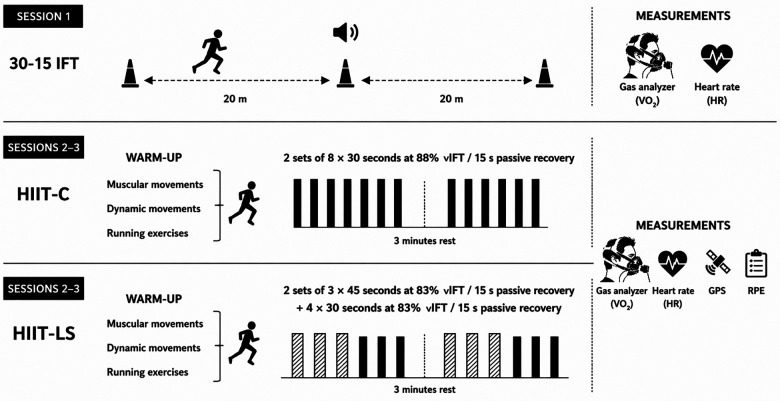
Study design. Session 1) 30-15 Intermittent Fitness Test (30-15 IFT). Pulmonary gas exchanges and heart rate were recorded. Sessions 2 and 3) A standardised warm-up based on movements and running drills was performed prior to each high-intensity interval training session. Constant work-interval high-intensity interval training (HIIT-C) consisted of 2 × 8 repetitions of 30 s at 88% of the maximal running velocity at the 30-15 IFT (vIFT). Long-start high-intensity interval training (HIIT-LS) consisted of 2 × 7 repetitions (3 × 45 s followed by 4 × 30 s) at 83% vIFT. Pulmonary gas exchanges, heart rate, and external load were recorded during HIIT sessions. Rating of perceived exertion (RPE) was collected 30 min after each HIIT session.

Participants completed three sessions over a ten-day period (Figure 1), with a minimum of one rest day between each session. All sessions were scheduled within a three-week in-season window during which no championship matches were played. Conditioning sessions replaced regular rugby-specific fitness training to maintain overall training load, and lower-body resistance training was suspended for the duration of the study period. Participants were instructed to arrive 90 min prior to each session in a rested and euhydrated state, with water provided upon arrival.

The first session comprised the 30-15 Intermittent Fitness Test (30-15IFT), used to assess aerobic fitness and determine the velocity at intermittent fitness test (vIFT) and V˙O_2_peak (29). The test consisted of 30 s shuttle runs over a 40 m course, interspersed with 15 s of passive recovery. Initial velocity was set at 8 km h^−1^ and increased by 0.5 km h^−1^ per stage, with pacing guided by auditory signals every 20 m. The test was terminated when a participant failed to reach the 3 m marker on three occasions within a single stage, and the running velocity corresponding to the last fully completed stage was recorded as vIFT. Each session was supervised by experienced strength and conditioning coaches, with standardised verbal encouragement provided throughout.

In the second and third sessions, participants completed the HIIT-C and HIIT-LS protocols using a randomized counterbalanced crossover design. Participants were randomly allocated to one of two protocol sequences (HIIT-C followed by HIIT-LS, or HIIT-LS followed by HIIT-C) prior to the start of the study to minimise potential order effects. The HIIT-C protocol (Figure 1) consisted of 2 sets of 8 intervals of 30 s at 88% vIFT (29), with 15 s of passive recovery between intervals and 3 min of passive recovery between sets. The HIIT-LS protocol (Figure 1) consisted of 2 sets of 7 intervals at 83% vIFT, with intervals 1–3 lasting 45 s and intervals 4–7 lasting 30 s, each separated by 15 s of passive recovery.

HIIT sessions were preceded by a standardised three-phase warm-up. Phase 1 comprised general mobility movements, including 10 squats, 10 lunges, and 10 hip thrusts. Phase 2 consisted of two sets of two squat jumps and two countermovement jumps. Phase 3 involved 10 running drills over 20 m, five accelerations over 50 m, and one 30 s run at 90% vIFT.

All HIIT sessions were conducted on the same outdoor synthetic turf surface between 16:00 and 18:30, under consistent environmental conditions (23.5 ± 1.9 °C, with a relative humidity of 61.7 ± 4.8%). Interval pacing was guided by auditory signals, and cones were individually spaced according to each participant's target velocity.

### 2.3 Outcome measures

#### 2.3.1 30-15 IFT measurements

HRpeak, V˙O_2_peak, and vIFT were determined from the 30-15IFT (29). Heart rate was recorded continuously using a Polar H10 heart rate monitor (Polar Electro Oy, Kempele, Finland) at a sampling frequency of 1 Hz. Pulmonary gas exchange was measured continuously at 15 s intervals using a portable metabolic analyser (Metamax 3B-R2, Cortex Biophysics, Leipzig, Germany), previously validated by Macfarlane & Wong (30). Participants wore the unit secured with a vest positioned on the thorax, with the device aligned over the clavicle to permit full arm mobility during running. An oronasal face mask (7,450 series V2, Hans Rudolph, Shawnee, KS, USA) was fitted to enable gas flow through a bidirectional digital turbine. Prior to each test, the flow sensor was calibrated using a 3 L syringe, and gas analysers were calibrated with ambient air and a reference gas mixture (15% O_2_, 5% CO_2_), in accordance with the manufacturer's recommendations. HRpeak was defined as the highest 1 s HR value recorded during the test, and V˙O_2_peak as the highest mean oxygen uptake recorded over a 30 s period prior to volitional exhaustion.

#### 2.3.2 HIIT session measurements

Heart rate was monitored continuously using the same Polar H10 heart rate monitor and sampling frequency (1 Hz) as described above. The time spent at or above 90% of peak heart rate (T ≥ 90% HRpeak) was defined as the cumulative duration during which heart rate equalled or exceeded 90% of HRpeak, as established from the 30-15IFT. Mean heart rate (HRmean) was calculated as the average HR recorded across all work intervals during the HIIT session, and the percentage of HRpeak (%HRpeak) was calculated as the average fraction of HRpeak during work intervals.

Pulmonary gas exchange was recorded continuously at 5 s intervals using the same metabolic analyser and calibration procedures as those employed during the 30-15IFT (Metamax 3B-R2, Cortex Biophysics, Leipzig, Germany). T ≥ 90%V˙O_2_peak was defined as the cumulative duration during which V˙O_2_ equalled or exceeded 90% of V˙O_2_peak. Mean oxygen uptake (V˙O_2_mean) was calculated as the average V˙O_2_ recorded across all work intervals, and the percentage of V˙O_2_peak (%V˙O_2_peak) was the average fraction of V˙O_2_peak during work intervals. Mean minute ventilation (V˙Emean) and mean respiratory frequency (*f*Rmean) were similarly determined as the average values recorded across all work intervals during the HIIT session.

External load during each HIIT session was quantified using 10 Hz GPS devices (Vector S7, Catapult Innovations, Melbourne, Australia). Each unit was positioned between the scapulae in a sport-specific vest and activated 15 min prior to data collection in accordance with the manufacturer's guidelines to ensure signal quality. Signal quality was confirmed by a mean horizontal dilution of precision (HDOP) of 0.75 ± 0.07 and a mean of 15.2 ± 0.5 connected satellites. To minimise inter-unit variability, the same device was assigned to each participant across all sessions.

Total distance was defined as the total metres covered during the session. Running distance was defined as the distance covered at speeds exceeding 7 km h^−1^, a threshold recognised as the transition from walking to running (31). High-speed running distance was defined as the distance covered at speeds exceeding 16 km h^−1^ (31). Mechanical work distance was defined as the total distance covered during acceleration and deceleration phases exceeding 2 m s^−1^ (32).

Thirty minutes after each HIIT session, participants rated their perceived exertion using the Borg CR10 scale (33).

### 2.4 Statistical analysis

Statistical analysis was structured in two phases. The first phase comprised a global comparison between protocols, in which mean values calculated over the entire duration of each session were evaluated to provide an overall assessment of protocol differences. Time-point comparisons were subsequently performed by analysing the protocols at discrete intervals, both at the set and half-set levels, with each set divided into two 3 min segments (0–3 min and 3–6 min). Equivalent sets were compared across protocols (e.g., Set 1 of HIIT-C vs. Set 1 of HIIT-LS; Set 2 of HIIT-C vs. Set 2 of HIIT-LS). All data are presented as means ± standard deviation (SD), along with mean differences and corresponding 95% confidence intervals (95% CI). Data normality was assessed using the Shapiro–Wilk test. Depending on the distribution of the data, either a paired t-test or a Wilcoxon signed-rank test was used to compare protocols. Statistical significance was set at *p* < 0.05.

In addition to inferential statistics, mean differences (HIIT-LS−HIIT-C) and their associated 95% confidence intervals were calculated to quantify the magnitude and precision of between-protocol effects. Given the crossover design and the relatively small sample size, standardized effect sizes (e.g., Cohen's d) were not reported, as estimation-based metrics (mean differences and confidence intervals) were considered more appropriate and more interpretable for the present analysis framework. Additionally, no corrections for multiple comparisons were applied across the physiological, external-load, and perceptual variables examined, in order to avoid inflating the risk of Type II error in this exploratory context.

Analyses were conducted across physiological variables (T ≥ 90%V˙O_2_peak, V˙O_2_mean, mean %V˙O_2_peak, HRmean, mean %HRpeak, V˙Emean, and *f*Rmean), external load variables (total distance, distance covered at or above 7 km h^−1^, distance covered at or above 16 km h^−1^, and mechanical work distance), and the perceptual variable RPE. All statistical analyses were performed using JASP (version 0.19.3; JASP Team, Amsterdam, Netherlands) and Microsoft Excel (Microsoft Corporation, Redmond, WA, USA).

## 3 Results

### 3.1 Physiological responses

**Figure 2:**
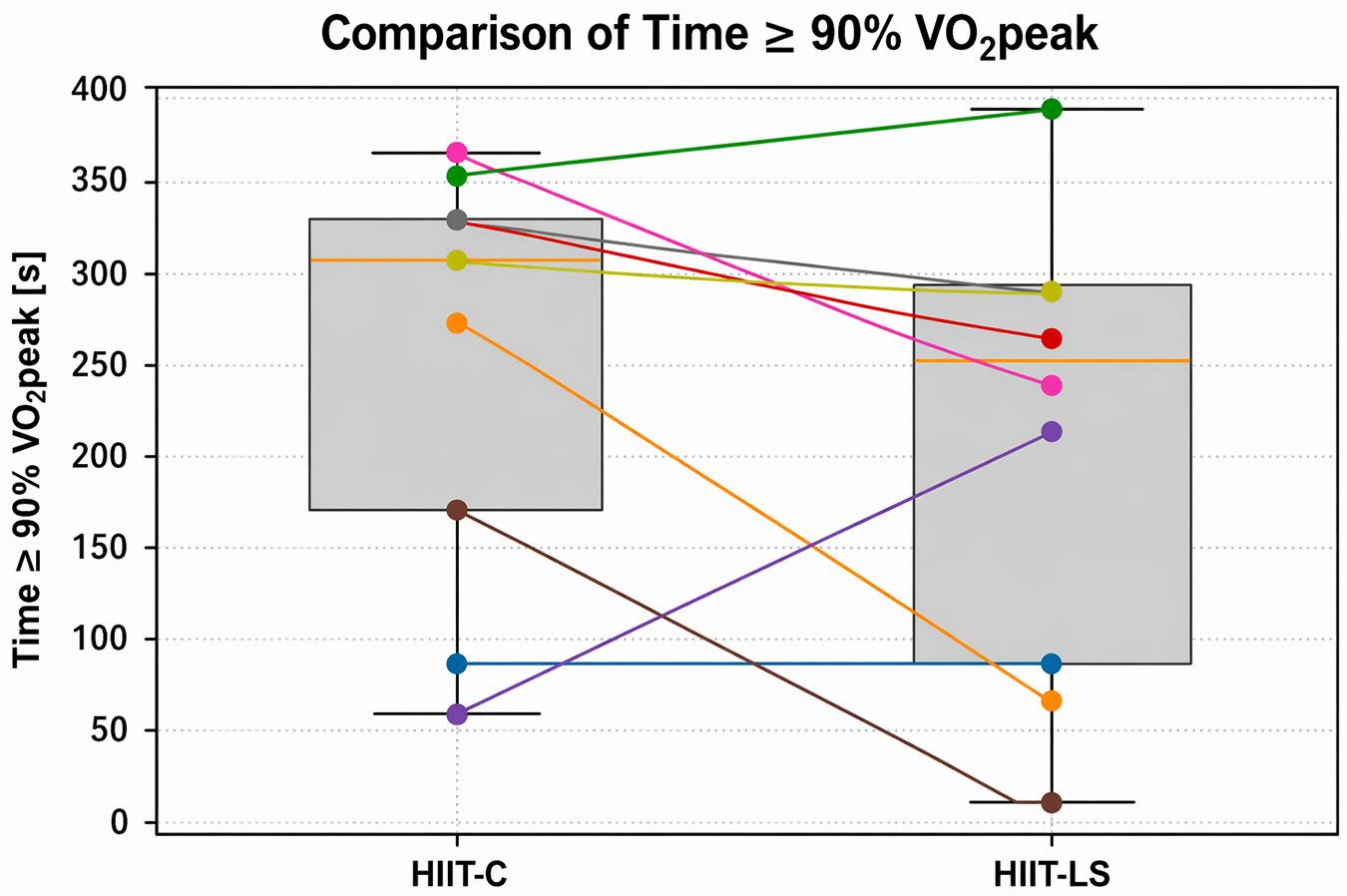
Time spent at or above 90% of peak oxygen uptake for HIIT-C and HIIT-LS during the total session. Individual trends and boxplots display the median, interquartile range, individual data points, minimum and maximum values. HIIT-C, constant work-interval high-intensity interval training; HIIT-LS, long-start high-intensity interval training; V˙O_2_peak, peak oxygen uptake; T ≥ 90%V˙O_2_peak, time spent at or above 90% of peak oxygen uptake.

**Figure 3:**
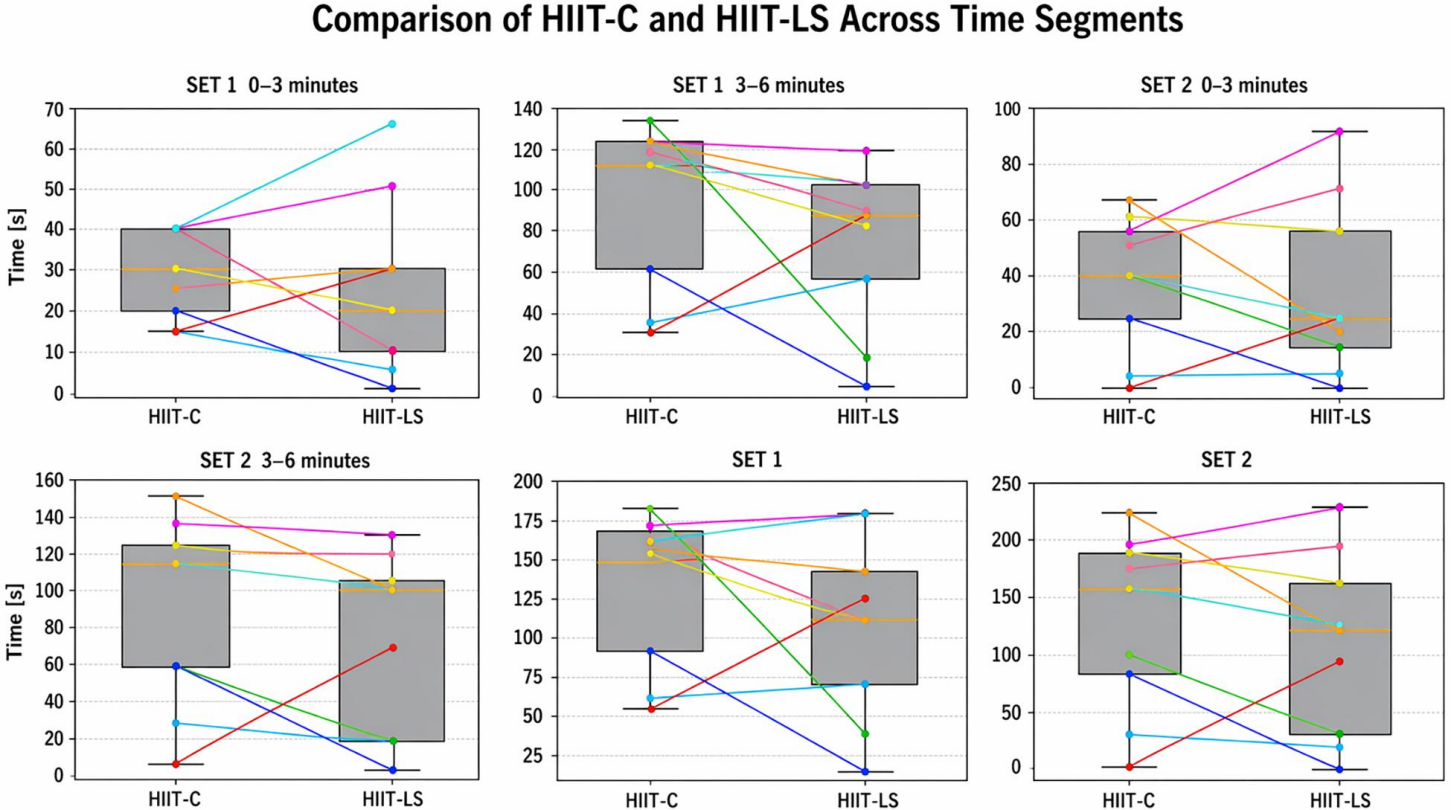
Time spent at or above 90% of peak oxygen uptake for HIIT-C and HIIT-LS across sets and time segments. Data are presented for the 0–3 min segment (intervals 1–4 for HIIT-C and intervals 1–3 for HIIT-LS) and the 3–6 min segment (intervals 5–8 for HIIT-C and intervals 4–7 for HIIT-LS) within Sets 1 and 2, as well as for the total duration of each set. HIIT-C, constant work-interval high-intensity interval training; HIIT-LS, long-start high-intensity interval training; V˙O_2_peak, peak oxygen uptake; T ≥ 90%V˙O_2_peak, time spent at or above 90% of peak oxygen uptake.

Physiological responses are summarised in Table 2 and illustrated in Figures 2, 3. No significant differences were observed between HIIT-LS and HIIT-C for V˙O_2_mean, mean %V˙O_2_peak, T ≥ 90%V˙O_2_peak, V˙Emean, *f*Rmean, or heart rate measures (HRmean, %HRpeak, T ≥ 90% HRpeak) across Set 1, Set 2, or the whole session. The only exception was V˙Emean during the second segment of Set 1 (3–6 min), which was higher in HIIT-C compared to HIIT-LS (mean paired difference = 5.4 L min^−1^; 95% CI: 1.64–9.13, *p* = 0.011).

**Table 2:** Comparison of physiological parameters between HIIT-C and HIIT-LS protocols.

Time segment	Variable	HIIT-C	HIIT-LS	Δ	*p*-value
SET 1	V˙O_2_mean (mL kg^−1^ min^−1^)	43.2 ± 3.4 (40.6–45.9)	42.5 ± 3.9 (39.4–45.5)	−0.8	0.229
	Mean %V˙O_2_peak	82.8 ± 2.8 (80.6–84.9)	81.2 ± 3.3 (78.7–83.8)	−1.6	0.200
	HRmean (bpm)	175 ± 9 (168–181)	174 ± 7 (169–180)	−1	0.910
	Mean %HRpeak	90.3 ± 1.8 (88.9–91.8)	90.6 ± 1.9 (89.2–92.1)	+0.3	0.710
	T ≥ 90%HRpeak (s)	249 ± 45 (214–284)	250 ± 42 (218–282)	+1	0.951
	V˙Emean (L min^−1^)	122.4 ± 18.2 (108.4–136.4)	120.4 ± 18.1 (106.5–134.3)	−2	0.307
	*f*_R_mean (cycles min^−1^)	45 ± 8.1 (38.9–51.2)	44.6 ± 6.9 (39.4–49.9)	−0.4	0.263
SET 1 (0–3 min)	V˙O_2_mean (mL kg^−1^ min^−1^)	39.9 ± 3.5 (37.2–42.6)	39.2 ± 3.4 (37.3–42.5)	+0.7	0.956
	Mean %V˙O_2_peak	76.3 ± 2.6 (74.3–78.3)	76.4 ± 3.2 (73.9–78.9)	+0.1	0.931
	HRmean (bpm)	167 ± 9 (160–174)	168 ± 6 (163–173)	+1	0.493
	Mean %HRpeak	86.3 ± 2.4 (84.4–88.1)	87.2 ± 2.1 (85.6–88.3)	+0.9	0.341
	T ≥ 90%HRpeak (s)	75 ± 39 (45–105)	78 ± 30 (55–101)	+3	0.820
	V˙Emean (L min^−1^)	107.3 ± 18.2 (93.3–121.3)	108.7 ± 17.2 (95.5–122)	+1.4	0.561
	*f*_R_mean (cycles min^−1^)	41.7 ± 8.1 (35.4–47.9)	42.8 ± 6.3 (38–47.6)	+1.1	0.263
SET 1 (3–6 min)	V˙O_2_mean (mL kg^−1^ min^−1^)	46.6 ± 3.4 (43.9–49.2)	45 ± 4.6 (41.5–48.5)	−1.6	0.063
	Mean %V˙O_2_peak	89.2 ± 3.7 (86.4–92)	86.1 ± 4 (83–89.1)	−3.1	0.229
	HRmean (bpm)	183 ± 8 (176–189)	181 ± 7.1 (176–187)	−2	0.162
	Mean %HRpeak	94.4 ± 1.6 (93.2–95.7)	94.1 ± 2 (92.6–95.6)	−0.3	0.623
	T ≥ 90%HRpeak (s)	174 ± 8 (168–180)	172 ± 13 (162–182)	−2	1
	V˙Emean (L min^−1^)	137.4 ± 18.5 (123.2–151.7)	132 ± 19.6 (117–147.1)	−5.4	0.011
	*f*_R_mean (cycles min^−1^)	48.4 ± 8.2 (42.1–54.7)	47.1 ± 6.9 (41.7–52.4)	−1.3	0.55
SET 2	V˙O_2_mean (mL kg^−1^ min^−1^)	43.7 ± 3.1 (41.4–46.1)	42.9 ± 4.4 (39.6–46.3)	−0.8	0.231
	Mean %V˙O_2_peak	83.8 ± 3.5 (81.1–86.5)	82.1 ± 4.7 (78.5–85.7)	−1.7	0.204
	HRmean (bpm)	180 ± 7 (175–186)	180 ± 6 (175–185)	0	0.716
	Mean %HRpeak	93.3 ± 1.5 (92.2–94.5)	93.5 ± 1.5 (92.3–94.6)	+0.2	0.806
	T ≥ 90%HRpeak (s)	298 ± 14 (288–309)	303 ± 24 (285–321)	+5	0.623
	V˙Emean (L min^−1^)	135.6 ± 16.9 (122.6–148.6)	134 ± 20.4 (117.7–149.1)	−1.6	0.458
	*f*_R_mean (cycles min^−1^)	51.8 ± 9.7 (44.4–59.3)	50.2 ± 8.1 (44–56.5)	−1.6	0.258
SET 2 (0–3 min)	V˙O_2_mean (mL kg^−1^ min^−1^)	40.8 ± 3.3 (38.3–43.4)	40.6 ± 4.4 (39.6–46.3)	−0.2	0.714
	Mean %V˙O_2_peak	83.8 ± 3.5 (81.1–86.5)	82.1 ± 4.7 (78.6–85.7)	−1.7	0.648
	HRmean (bpm)	174 ± 7.3 (168–180)	174 ± 6.2 (169–179)	0	0.994
	Mean %HRpeak	90 ± 1.6 (88.9–91.2)	90.4 ± 1.7 (89.1–91.8)	+0.4	0.628
	T ≥ 90%HRpeak (s)	118 ± 14 (108–129)	123 ± 23 (106–141)	+5	0.606
	V˙Emean (L min^−1^)	122.3 ± 17.3 (109–135.6)	122.7 ± 19.6 (107.7–137.8)	+0.4	0.887
	*f*_R_mean (cycles min^−1^)	48.3 ± 10 (40.6–56)	47.7 ± 7.8 (41.7–53.6)	−0.6	0.666
SET 2 (3–6 min)	V˙O_2_mean (mL kg^−1^ min^−1^)	46.6 ± 2.9 (44.4–48.9)	45.3 ± 4.6 (41.8–48.8)	−1.3	0.092
	Mean %V˙O_2_peak	89.4 ± 4 (86.3–92.5)	86.6 ± 5.1 (82.7–90.5)	−2.8	0.085
	HRmean (bpm)	187 ± 7.1 (181–192)	186 ± 6 (181–191)	−1	0.45
	Mean %HRpeak	96.6 ± 1.6 (95.3–98.0)	96.5 ± 1.4 (95.4–97.6)	−0.1	0.899
	T ≥ 90%HRpeak (s)	180 ± 0 (180–180)	180 ± 1 (179–180)	0	1
	V˙Emean (L min^−1^)	148.9 ± 17 (135.8–161.9)	144.1 ± 21.4 (127.6–160.5)	−4.8	0.111
	*f*_R_mean (cycles min^−1^)	55.4 ± 10 (47.9–63.1)	52.8 ± 8.6 (46.2–59.4)	−2.6	0.25
Total session	V˙O_2_mean (mL kg^−1^ min^−1^)	43.5 ± 3.2 (41–46)	42.7 ± 4.1 (39.5–45.9)	−0.8	0.203
	Mean %V˙O_2_peak	83.3 ± 3 (80.9–85.6)	81.6 ± 3.8 (78.7–84.6)	−1.7	0.175
	HRmean (bpm)	178 ± 8 (172–183)	177 ± 6 (172–182)	−1	0.773
	Mean %HRpeak	91.8 ± 1.5 (90.7–93)	92.1 ± 1.5 (90.9–93.3)	+0.3	0.4
	T ≥ 90%HRpeak (s)	548 ± 52 (508–587)	553 ± 56 (510–596)	+5	0.793
	V˙Emean (L min^−1^)	129 ± 17.3 (115.7–142.2)	126.9 ± 19.1 (112.2–141.6)	−2.1	0.342
	*f*_R_mean (cycles min^−1^)	48.4 ± 8.8 (41.7–55.2)	47.6 ± 7.2 (42–53.2)	−0.8	0.404

Data are presented as mean ± standard deviation with 95% confidence intervals in parentheses. The difference column (Δ) reflects HIIT-LS minus HIIT-C values. Statistical significance was set at *p* < 0.05. The significant *p*-value is shown in bold.

HIIT-C, constant work-interval high-intensity interval training; HIIT-LS, long-start high-intensity interval training; V˙O_2_mean, mean oxygen uptake; Mean %V˙O_2_peak, percentage of peak oxygen uptake; HRmean, mean heart rate; %HRpeak, percentage of peak heart rate; T ≥ 90%HRpeak, time spent at or above 90% of peak heart rate; V˙Emean, mean minute ventilation; *f*Rmean, mean respiratory frequency.

### 3.2 External load and perceived effort

GPS-derived external load variables are summarised in Table 3. Compared to HIIT-C, HIIT-LS elicited greater total distance (mean paired difference =  +63.9 m; 95% CI: 57.3–70.5, *p* < 0.01) and distance covered above 7 km h^−1^ mean paired difference =  + 70.9; 95% CI: 64.8–77.0; *p* < 0.01), lower mechanical work distance (mean paired difference = −37.4 m; 95% CI: −41.9 to −32.7, *p* < 0.01), and reduced perceived exertion (RPE) (mean paired difference = − 0.6 AU; 95% CI: −1.2 to −0.1; *p* = 0.048). No significant differences were observed for distance covered above 16 km h^−1^.

**Table 3:** Comparison of external load and perceptual responses between HIIT-C and HIIT-LS protocols.

	HIIT-C	HIIT-LS	Δ	*p*-value
Total Distance (m)	2,207.3 ± 105.3	2,271.2 ± 107.4	+63.9	***p*** **<** **0.01**
Distance ≥ 7 km h^−1^ (m)	2,086.5 ± 110.4	2,157.4 ± 116.4	+70.9	***p*** **<** **0.01**
Distance ≥ 16 km h^−1^ (m)	1,427.8 ± 599.9	1,326.4 ± 583.9	−101.4	0.098
MW distance (m)	149.3 ± 16.4	111.9 ± 11.1	−37.4	***p*** **<** **0.01**
RPE (0–10)	8.3 ± 0.7	7.7 ± 0.7	−0.6	**0.048**

Data are presented as mean ± standard deviation. The difference column (Δ) reflects HIIT-LS minus HIIT-C values. Statistical significance was set at *p* < 0.05. The significant *p*-value is shown in bold.

HIIT-C, constant work-interval high-intensity interval training; HIIT-LS, long-start high-intensity interval training; Distance ≥ 7 km h^−1^, distance covered at speeds greater than 7 km h^−1^; Distance ≥ 16 km h^−1^, distance covered at speeds greater than 16 km h^−1^; MW distance, distance covered greater than 2 m s^−2^ during both acceleration and deceleration phases; RPE, rating of perceived exertion; AU, arbitrary units.

## 4 Discussion

To the authors' knowledge, the present study is the first to examine a long-start HIIT strategy in a team-sport context, using a running-based intensity prescription derived from the 30-15IFT within a short-interval training format. The aim of the present study was therefore to compare the physiological, perceptual, and external-load responses between HIIT-LS and HIIT-C in academy rugby union players. It was hypothesised that HIIT-LS would increase T ≥ 90%V˙O_2_peak without altering perceptual or external-load responses. Contrary to this hypothesis, HIIT-LS did not significantly increase T ≥ 90%V˙O_2_peak or mean %V˙O_2_peak compared to HIIT-C. However, HIIT-LS elicited lower RPE despite greater external load, suggesting that perceptual responses during intermittent exercise may be influenced more by exercise intensity than by total running volume alone.

HIIT is widely recognised as an effective strategy for improving aerobic fitness and V˙O_2_peak in team-sport athletes (18). Among the proposed mechanisms underlying these adaptations, accumulating time at or above 90%V˙O_2_peak has been considered a key stimulus for promoting central and peripheral aerobic adaptations (16, 25). Although extending the duration of the initial intervals in HIIT-LS may theoretically accelerate V˙O_2_ kinetics and facilitate earlier attainment of high oxygen uptake values, this potential benefit was likely offset by the lower running intensity prescribed during HIIT-LS (83% vs. 88% vIFT). Since oxygen uptake kinetics are strongly intensity-dependent, the lower exercise intensity may have reduced the metabolic demand required to rapidly achieve and sustain high fractions of V˙O_2_peak. This interpretation is supported by the small between-protocol differences observed in T ≥ 90%V˙O_2_peak (mean difference = − 43.9 s, 95% CI: −126.2 to 38.4), V˙O_2_mean (mean difference = − 0.8 mL kg^−1^ min^−1^, 95% CI: −2.1 to 0.5) and mean %V˙O_2_peak (mean difference = − 1.6%, 95% CI: −4.1 to 0.9), which suggest limited alterations in overall oxygen uptake between conditions. The latter marker is of particular relevance, as longer durations spent at a high %V˙O_2_peak have been associated with greater training-induced adaptations and improvements in endurance performance (19, 34). These findings are consistent with previous evidence demonstrating that relatively small increases in exercise intensity during short-interval HIIT can substantially increase time spent near V˙O_2_max in trained athletes (35), highlighting the strong sensitivity of oxygen uptake kinetics to even minor changes in prescribed intensity.

The present findings differ from previous studies conducted in endurance sports, where varied-parameter HIIT strategies, including fast-start and long-start approaches, generally increased T ≥ 90%V˙O_2_peak compared with constant-intensity protocols (20–25, 27, 28). Notably, a distinct approach, involving longer initial intervals followed by a progressive reduction in interval duration, has consistently been shown to elicit significantly greater T ≥ 90%V˙O_2_peak compared to both long-interval and short-interval conditions across two independent studies in cyclists (312 s vs. 179 s vs. 183 s, respectively) (27) and runners (579 s vs. 349 s vs. 167 s, respectively) (28). Although these studies share a conceptual basis with the present investigation by implementing longer initial intervals within HIIT sessions, key design differences, including exercise modality (cycling and running vs. team-sport running), athlete population (endurance-trained vs. rugby union players), and protocol structure limit direct comparison with the present findings and the physiological mechanisms underpinning these responses may not directly transfer to intermittent team-sport contexts such as rugby union. The repeated accelerations, decelerations, and directional changes characteristic of intermittent running may alter oxygen uptake kinetics and reduce the effectiveness of pacing strategies initially developed for continuous endurance exercise. Furthermore, unlike endurance athletes, rugby union players may exhibit greater neuromuscular and anaerobic contributions during intermittent exercise, potentially limiting the capacity to sustain elevated oxygen uptake despite longer work intervals (1, 36).

Contrary to expectations, RPE was significantly lower following HIIT-LS than HIIT-C despite greater total external load. This finding contrasts with previous observations reporting greater perceived exertion during varied-parameter HIIT in rugby union players (26). The lower running intensity prescribed during HIIT-LS likely reduced neuromuscular strain, metabolite accumulation, and cardiorespiratory stress per unit of work, thereby attenuating afferent feedback associated with effort perception. In addition, the longer initial intervals may have promoted a more progressive physiological adjustment at exercise onset, potentially reducing the abrupt metabolic perturbations commonly associated with repeated short high-intensity efforts. Together, these findings suggest that perceptual responses during intermittent HIIT may be more closely related to instantaneous exercise intensity than to total running volume alone.

Nevertheless, the lower prescribed intensity used during HIIT-LS represents an important limitation when interpreting the present findings. The intensity reduction was intentionally implemented to facilitate completion of the longer initial intervals while maintaining the ecological validity and practical applicability of the protocol in rugby union settings. However, it remains difficult to determine whether the observed perceptual and physiological differences were attributable to the long-start interval structure itself or simply to the lower running intensity prescribed during HIIT-LS. Higher exercise intensities have previously been associated with greater motor unit recruitment and more homogeneous activation of the quadriceps femoris musculature (37, 38), potentially increasing type II muscle fibre recruitment. Given the lower mechanical efficiency and higher oxygen demand of type II fibres, these mechanisms may partly explain the greater oxygen uptake responses typically observed during higher-intensity interval exercise (39).

Heart rate responses were similar between protocols across all variables, with mean differences close to zero and confidence intervals overlapping zero, despite differences in perceptual and external-load measures. This finding highlights the limited sensitivity of heart rate to detect subtle variations in metabolic stress during short-interval HIIT. Due to its slower kinetic response and cumulative cardiovascular drift, heart rate may primarily reflect overall cardiovascular strain rather than transient fluctuations in muscular oxygen demand during intermittent exercise (14). Comparable findings have been reported in previous studies employing varied-parameter HIIT strategies in endurance athletes (28, 34).

From an external-load perspective, HIIT-LS induced greater total distance and running distance ≥7 km h^−1^ but lower mechanical work distance compared with HIIT-C, while no clear difference was observed for high-speed running distance ≥16 km h^−1^. The greater total distance and distance ≥7 km h^−1^ observed during HIIT-LS were primarily driven by the longer work intervals despite the lower prescribed intensity. However, the absence of differences in high-speed running distance suggests that the additional locomotor load accumulated during HIIT-LS was mainly performed at submaximal running velocities. This distinction may partly explain why the greater external load did not translate into greater oxygen uptake responses. In addition, caution is warranted when interpreting GPS-derived locomotor variables during short-interval exercise due to the known limitations of GPS technology in detecting rapid velocity fluctuations and changes of direction (40).

Furthermore, the absence of complementary physiological measurements such as blood lactate concentration, muscle oxygenation, or neuromuscular fatigue markers limits the ability to fully elucidate the mechanisms underlying the observed responses. In addition, although the sample size was determined *a priori* and fell within the estimated range required for adequate statistical power, the relatively small sample remains a limitation. However, studies involving detailed physiological assessment during repeated HIIT protocols in trained athletes commonly include limited sample sizes due to the substantial logistical and methodological demands associated with such experimental designs.

Consequently, the independent effect of the long-start strategy cannot be fully isolated from the influence of exercise intensity. The lower running intensity prescribed during HIIT-LS was intentionally selected to ensure completion of the longer initial intervals while maintaining the ecological validity and practical applicability of the protocol in rugby union settings. This prescription approach follows the individualised HIIT intensity recommendations derived from the 30-15IFT, in which vIFT serves as a reference speed to account for inter-individual differences in aerobic capacity, anaerobic speed reserve, and change-of-direction abilities (29). However, because exercise intensity is a major determinant of oxygen uptake kinetics, neuromuscular recruitment, biomechanical stress, and perceptual responses during HIIT, it remains unclear whether the lower RPE and altered external-load responses observed during HIIT-LS were attributable to the interval configuration itself or simply to the reduced relative intensity. Therefore, the present findings should be interpreted cautiously, as reflected in the width of several confidence intervals. Future studies should directly compare long-start and constant-intensity HIIT protocols using matched exercise intensities to better isolate the specific effects of interval structure manipulation.

### 4.1 Practical implications

From a practical standpoint, HIIT-LS did not result in meaningful increases in time spent at high oxygen uptake compared with HIIT-C in young elite male rugby union players. Therefore, when the primary objective is to maximise aerobic stimulus and time spent near V˙O_2_peak, practitioners may preferentially select traditional short-interval HIIT formats prescribed at higher relative running intensities.

However, the lower perceived exertion observed during HIIT-LS despite greater external load may provide meaningful practical advantages for coaches working in intermittent team-sport settings. Specifically, HIIT-LS may represent a useful strategy when the goal is to accumulate running volume and mechanical load while limiting perceptual stress and potentially reducing overall psychophysiological fatigue. This may be particularly relevant during pre-season phases focused on progressive aerobic conditioning, during congested competition schedules where recovery management is critical, or potentially during return-to-play processes in which gradual re-exposure to running load is required, though such applications would require further clinical and applied validation before firm recommendations can be made.

In applied settings, coaches may therefore use HIIT-LS as a transitional conditioning strategy positioned between lower-intensity aerobic conditioning and more demanding high-intensity interval formats. The progressive reduction in interval duration may also facilitate pacing regulation and improve session tolerability in athletes with lower training readiness or elevated fatigue levels. In addition, prescribing running intensity relative to vIFT obtained from the 30-15IFT provides an ecologically valid and easily implementable approach for individualising training load in rugby union environments.

Nevertheless, the present findings should be interpreted cautiously due to the relatively small sample size and the lower running intensity prescribed during HIIT-LS. Future research should directly compare HIIT-LS, HIIT-C, and HIIT-FS protocols using matched running intensities and longitudinal training interventions to better determine their respective effects on aerobic adaptations, recovery, and performance outcomes in team-sport athletes.

## 5 Conclusion

In young elite male academy rugby union players, the HIIT-LS protocol did not elicit greater T ≥ 90%V˙O_2_peak or mean %V˙O_2_peak compared to HIIT-C, despite involving a greater total exercise duration and external load. However, HIIT-LS was associated with significantly lower perceived exertion, while heart rate responses remained comparable between protocols.

These findings suggest that, within rugby union conditioning programs, HIIT-LS may not represent the most effective strategy when the primary objective is to maximise aerobic stimulus and time spent near V˙O_2_peak. In such contexts, traditional constant-intensity short-interval HIIT prescribed at higher relative intensities may remain preferable. Nevertheless, the lower perceptual strain observed during HIIT-LS despite greater running volume may provide meaningful practical advantages for practitioners seeking to progressively accumulate locomotor and mechanical load while limiting psychophysiological stress.

Consequently, HIIT-LS may represent a useful conditioning strategy during specific phases of the rugby union training process, particularly during pre-season preparation, periods of fixture congestion, or return-to-play contexts where progressive load management is a priority, though such applications should be considered exploratory given the limitations of the present design. In applied settings, HIIT-LS may therefore serve as a transitional aerobic conditioning method between lower-intensity aerobic training and more demanding high-intensity interval formats.

The present study provides novel insight into the acute physiological, perceptual, and external-load responses to a long-start interval training strategy in a team-sport context using a running-based intensity prescription aligned with the intermittent demands of rugby union. However, the findings should be interpreted cautiously, as the two HIIT protocols differed not only in interval structure but also in prescribed running intensity (83% vs. 88% vIFT). Consequently, it is not possible to fully isolate whether the observed differences in perceptual and external-load responses were attributable to the long-start strategy itself or to the lower exercise intensity prescribed during HIIT-LS. Future studies should therefore compare HIIT-LS and HIIT-C using matched exercise intensities and longitudinal training designs to better isolate the specific effects of interval structure manipulation across different team sports, training phases, and athlete populations.

## Data Availability

The raw data supporting the conclusions of this article will be made available by the authors, without undue reservation.
